# Negligible role of TRAIL death receptors in cell death upon endoplasmic reticulum stress in B-cell malignancies

**DOI:** 10.1038/s41389-023-00450-w

**Published:** 2023-02-08

**Authors:** Francesca Favaro, Demi Both, Ingrid A. M. Derks, Marcel Spaargaren, Cristina Muñoz-Pinedo, Eric Eldering

**Affiliations:** 1 Amsterdam UMC location University of Amsterdam, Dept. of Experimental Immunology, Meibergdreef 9, Amsterdam, the Netherlands; 2 Amsterdam institute for Infection and Immunity, Cancer Immunology, Amsterdam, the Netherlands; 3 Cancer Center Amsterdam, Cancer Biology and Immunology, Amsterdam, the Netherlands; 4 Lymphoma and Myeloma Center Amsterdam (LYMMCARE), Amsterdam, The Netherlands; 5 Preclinical and Experimental Research in Thoracic Tumors (PReTT), Molecular Mechanisms and Experimental Therapy in Oncology Program (Oncobell), Bellvitge Biomedical Research Institute (IDIBELL), L’Hospitalet de Llobregat, 08908 Barcelona, Spain; 6 Amsterdam UMC location University of Amsterdam, Dept. of Pathology, Meibergdreef 9, Amsterdam, the Netherlands

**Keywords:** Cancer microenvironment, Apoptosis, Stress signalling

## Abstract

Impairments in protein folding in the endoplasmic reticulum (ER) lead to a condition called ER stress, which can trigger apoptosis via the mitochondrial or the death receptor (extrinsic) pathway. There is controversy concerning involvement of the death receptor (DR)4 and DR5-Caspase-8 –Bid pathway in ER stress-mediated cell death, and this axis has not been fully studied in B-cell malignancies. Using three B-cell lines from Mantle Cell Lymphoma, Waldenström’s macroglobulinemia and Multiple Myeloma origins, we engineered a set of CRISPR KOs of key components of these cell death pathways to address this controversy. We demonstrate that DR4 and/or DR5 are essential for killing via TRAIL, however, they were dispensable for ER-stress induced-cell death, by Thapsigargin, Brefeldin A or Bortezomib, as were Caspase-8 and Bid. In contrast, the deficiency of Bax and Bak fully protected from ER stressors. Caspase-8 and Bid were cleaved upon ER-stress stimulation, but this was DR4/5 independent and rather a result of mitochondrial-induced feedback loop subsequent to Bax/Bak activation. Finally, combined activation of the ER-stress and TRAIL cell-death pathways was synergistic with putative clinical relevance for B-cell malignancies.

## Introduction

The endoplasmic reticulum (ER) mediates the folding of proteins destined for the plasma membrane and secretion. Disruptions during this process can lead to the accumulation of unfolded proteins, a condition known as ER stress, which activates the unfolded protein response (UPR) to promote cellular repair. In the case of unmitigated stress, the UPR triggers an apoptotic cascade [1–3]. Together with Inositol-Requiring Enzyme 1 (IRE1), protein kinase R-like kinase (PERK) is the main sensor of this response, which induces phosphorylation of eukaryotic translation-initiation factor 2α (eIF2α). This suppresses *de novo* protein synthesis but allows translation of selected genes, such as chaperones and also the transcription factors ATF4 and CHOP, for restoration of cellular homeostasis. If the cells do not recover, however, ATF4 and CHOP promote cell death by upregulating death-related genes like the TRAIL receptors TNFRSF10B (gene name for death receptor 5 - DR5) [4, 5], TNFRSF10A (death receptor 4 – DR4) [6], Noxa [7, 8] and BIM [9, 10].

B-cell malignancies are characterized by uncontrolled B-cell proliferation and immunoglobulin production, which make them operate at high basal activity of the ER [11–13]. Moreover, the UPR is required for B-cells to differentiate into plasma cells, as well as in establishing hematologic malignancies [14–16]. For instance, in multiple myeloma (MM), the characteristic excessive production of immunoglobulins (paraprotein or M-protein) by the malignant plasma cells causes ER-stress which renders these cells highly dependent upon UPR-mediated cell survival [17]. Accordingly, a correlation was found between disease state and UPR activity, which pointed to the UPR as a potent drug target [17, 18]. Indeed, proteasome inhibitors like Bortezomib, which will hyper-stress the ER and tip the balance towards UPR-mediated cell death, display clinical efficacy with improved time without progressive disease in patients with MM, mantle cell lymphoma (MCL) or Wäldenstrom macroglobulinemia [19, 20]. However, the treatment showed strong systemic side effects and regularly displayed drug-resistance relapse in the long-term [3, 21]. This could be explained by the elevated susceptibility of these cells to ER stress-induced apoptosis [22, 23]. To improve treatment of these diseases, it would be necessary to mechanistically investigate the cell death responses upon ER stress induction.

Programmed cell death, or apoptosis, is generally divided in two interconnected cascades. The intrinsic, or mitochondrial, pathway that relies on Bax and/or Bak to permeabilize the mitochondrial membrane; with subsequent cytochrome c release and Caspase-9 activation. The extrinsic pathway is characterized by triggering of tumor necrosis factor (TNF) receptor family members (so-called death receptors) followed by Caspase-8 activation. Extrinsic apoptosis signalling varies between type I or type II cells, which differ in their requirement of the Bid-Bax/Bak axis activity to process the apoptotic signal [24, 25].

Several years ago, it was reported that DRs and the Caspase-8/Bid pathway were involved in executing apoptosis upon ER stress in cancer cells [4, 5, 26]. Subsequently, the contribution and requirement of DRs in ER stress was more widely studied and has met with some controversy, as their role is possibly cell-type dependent [4–6, 10, 27]. DR5 especially is reported to be important in tipping the balance towards the apoptotic fate, because its levels are tightly regulated by the UPR [28]. In these circumstances, DR5 activity is not only dependent on binding TRAIL ligand, but it can be directly activated by the accumulation of misfolded proteins, which in turn induces the oligomerization of DR5 and assembling of the death-inducing signalling complex (or DISC) to trigger apoptosis [27]. As it has been suggested [29] that this mechanism could be cell-type specific but hitherto left unexplored in B-cell malignancies, we aimed to delineate the role of both the death receptors (DR4 and DR5) in triggering cell death upon ER stress in distinct B-cell cancer lines.

## Results

### ER stress cell death sensitivities in diverse B-cell malignancy cell lines

**Fig. 1:**
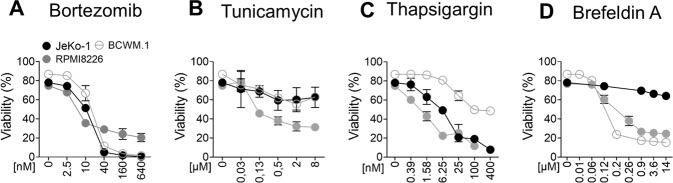
Distinct ER stress cell death sensitivities in B-cell malignancy cell lines. **A**–**D** JeKo-1, RPMI8226 and BCWM.1 cells were plated at 0.4×10^6^ cells/ml with the indicated drugs and doses for 24 hours. Viability was measured via FACS using DiOC6 and ToPro-3 staining. DiOC6+ and ToPro-3- cells were considered viable. Results are shown as Mean ± SEM (*N* = 3).

To investigate ER stress-mediated cell death in B-cell malignancies, we studied three different cell lines: a mantle cell lymphoma (JeKo-1), a Wäldenstrom macroglobulinemia (BCWM.1) and a multiple myeloma (RPMI8226). Various drugs with distinct actions of triggering ER stress were applied; Thapsigargin as inhibitor of calcium pump in the ER, Tunicamycin as inhibitor of N-glycosylation of proteins, Bortezomib as proteasome inhibitor and Brefeldin A that blocks ER-to-Golgi trafficking. While all three cell lines were similarly sensitive to Bortezomib (Fig. 1A), treatment with Tunicamycin showed no strong cell death induction except for moderate effects on RPMI8226 cells (Fig. 1B). Thapsigargin instead displayed similar viability curves on JeKo-1 and RPMI8226 cells but was less effective on BCWM.1 cells (Fig. 1C). Finally, Brefeldin A exhibited a limited efficacy on JeKo-1 cells while RPMI8226 and BCWM.1 cells responded alike on a dose dependent manner (Fig. 1D). In summary, each B-cell malignancy cell line displayed a distinct pattern of sensitivity to ER stress drugs.

### Death receptors are differentially expressed in B-cell malignancy cell lines

**Fig. 2:**
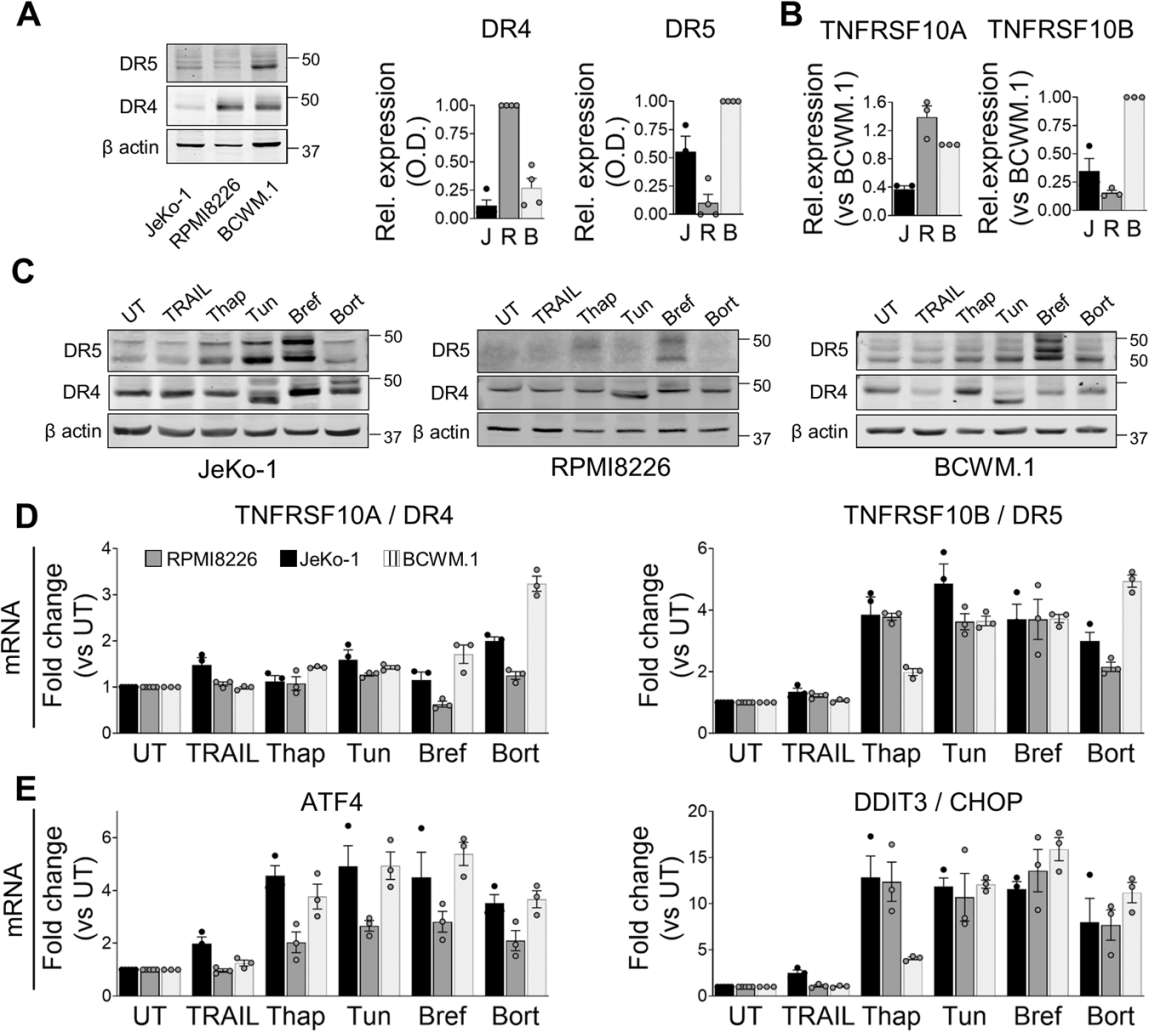
Expression of DR4/5 in cell lines upon TRAIL and ER-stress treatment. **A** JeKo-1, RPMI8226 and BCWM.1 cells were plated at 0.4×10^6^ cells/ml. After 24 hours incubation cells were collected and proteins extracted. DR4 and DR5 were measured on western blot using β-actin as loading control. A representative western blot is shown. Quantifications of DR4 and DR5 were performed using ImageJ. Results are shown normalized to their β-actin and as Mean of Relative expression compared to RPMI8226 for DR4 expression, and compared to BCWM.1 for DR5 expression (*N* = 3). J for JeKo-1 cells, R for RPMI8226 cells, B for BCWM.1 cells. **B** As in A but for mRNA. Transcripts of DR4 (TNFRSF10A) and DR5 (TNFRSF10B) were measured and normalized to TBP as housekeeping gene. Relative expression represented as Mean ± SEM, and normalized to BCWM.1 value. **C** JeKo-1, RPMI8226 and BCWM.1 cells were plated at 0.5×10^6^ cells/ml and incubated for 24 hours in media containing Q-VD (5 µM) with TRAIL or ER stress inducers at the following concentrations: JeKo-1 TRAIL 14 ng/ml, Thapsigargin (Thap) 100 nM, Tunicamycin (Tun) 500 nM, Brefeldin A (Bref) 228 µM and Bortezomib (Bort) 100 nM; for RPMI8226 TRAIL 14 ng/ml, Thap 200 nM, Tun 500 nM, Bref 1,8 µM and Bort 9 nM; and for BCWM.1 TRAIL 200 ng/ml, Thap 100 nM, Tun 2000 nM, Bref 2,2 µM and Bort 40 nM. Expression of DR4 and DR5 was measured, using β-actin as loading control. Representative western blots are shown for JeKo-1, RPMI8226 and BCWM.1 cells respectively. **D-E** As for C, cells were incubated with the indicated drugs and RNA was isolated. Levels of DR4 (TNFRSF10A), DR5 (TNFRSF10B), ATF4 and CHOP (DDIT3) mRNA was measured by qPCR. Results were normalized to TBP as housekeeping gene and shown as Fold change Mean ± SEM (*N* = 3).

In order to investigate a role for DRs in ER-stress in B-cell lines, we first characterized their expression at basal condition and under ER stress induction using concentrations based on the dose-response curves. The three B-cell malignancy cell lines showed different basal expressions of DR4 and DR5, both at protein (Fig. 2A) and mRNA levels (Fig. 2B). RPMI8226 cells displayed only DR4 expression, JeKo-1 cells expressed mainly DR5, and BCWM.1 cells displayed high levels of both DR4 and DR5. Super-killer TRAIL was taken as positive control for DR signalling and showed strong cell death sensitivity in JeKo-1 and RPMI8226 cells (Fig. S1A). To assess DR4/5 expression upon ER stress, the three cell lines were treated with Thapsigargin, Tunicamycin, Brefeldin A and Bortezomib at relatively high concentrations (cell death measurements in Fig. S1B), showing mild transcriptional and post-transcriptional regulation of DR4 (Fig. 2C, D and Fig. S1C). Treatment with Tunicamycin, which inhibits post-translational N-glycosylation, reduced the molecular weight of DR4 protein (Fig. 2C). In contrast, DR5 exhibited strong transcriptional increase upon treatment of all the ER stress drugs (Fig. 2D) and at the post-transcriptional level only upregulation under Brefeldin A in RPMI8226, JeKo-1 and BCWM.1 cell lines (Fig. 2C, D and Fig. S1C). DR5 was also robustly induced at the protein level upon Tunicamycin treatment but only in the JeKo-1 cell line (Fig. 2C and Fig. S1C). Expression levels of ATF4 and CHOP confirmed the induction of ER stress in all cell lines (Fig. 2E), by all ER stress drugs but not upon TRAIL stimulation. In cells derived from solid cancers, inhibition of protein synthesis by the UPR leads to decrease of cFLIP and Mcl-1 [2, 8, 30], but this was not observed in the cell lines studied here (Fig. S1D).

### Death receptors are essential proteins for TRAIL-induced cell death but not for ER stress-cell death induction

**Fig. 3:**
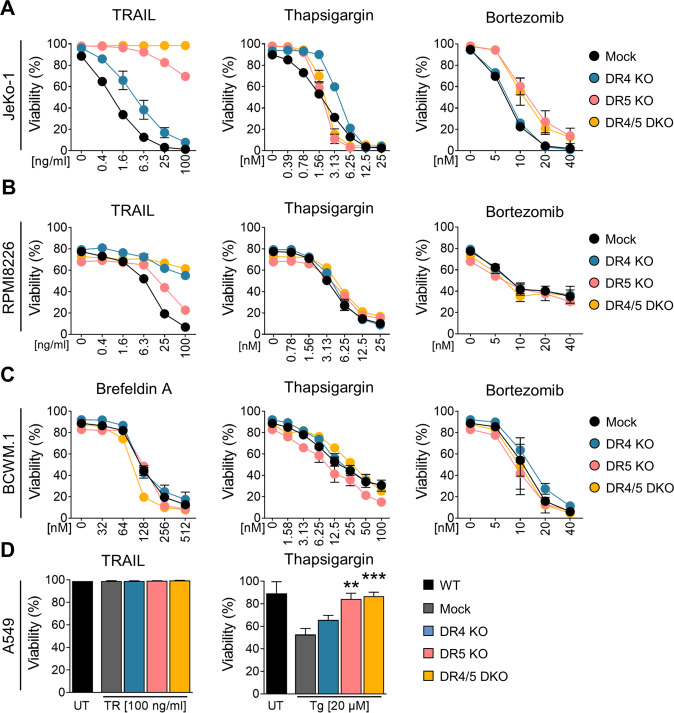
Consequences of DR4/5 gene ablation for TRAIL and ER-stress cell death responses. **A** JeKo-1 Mock, DR4 KO (pool), DR5 KO (clonal population) and DR4/5 DKO (clonal population) cells were incubated with TRAIL or Thapsigargin for 48 hours and with Bortezomib for 24 hours. Cells were then collected and viability was measured via FACS using DiOC6 and ToPro-3 staining. Analyses were performed selecting cells for DiOC6+ and ToPro-3-. Results are shown as Mean ± SEM (*N* = 3). **B** Same as A but with RPMI8226 cells but for 24 hours. RPMI8226 DR4 KO and DR4/5 DKO are pool of KO cells, DR5 KO is a clonal population. Results are shown as Mean ± SEM (*N* = 3). **C** Same as A but with BCWM.1 cells and with Brefeldin A instead of TRAIL treatment. BCWM.1 DR4 and DR5 KOs are clonal populations, DR4/5 DKO is a pool of KO cells. Results are shown as Mean ± SEM (*N* = 3). **D** A549 WT, Mock, DR4 KO (pool), DR5 KO (clonal population) and DR4/5 DKO (clonal population) cells were incubated with normal media or media containing 100 ng/ml TRAIL (TR) or 20 µM Thapsigargin (Tg) for 48 hours. Cells were then collected and viability was measured via FACS using Propidium Iodide (PI) staining. Dead cells were selected as PI^+^ cells. Results are shown as Mean ± SEM (TRAIL *N* = 3 and Thapsigargin *N* ≥ 4). Asterisks denote significant differences with Thapsigargin-treated mock-transfected cells.

We next focused on the involvement of DR4 and DR5 in ER stress-mediated-cell death, by creating CRISPR/Cas9 KOs in JeKo-1, RPMI8226 and BCWM.1 cell lines (Fig. S2A-E). JeKo-1 cells, which expressed high basal levels of DR5 but lower DR4 (Fig. 2A), displayed strong dependency on DR5 on TRAIL-mediated death that was further strengthened when both receptors were knocked-out (Fig. 3A). A similar but opposite effect was seen in RPMI8226 cells which mostly relied on DR4 upon TRAIL stimulation (Fig. 3B), in accordance also with their basal expression level (Fig. 2A, B). Notably, BCWM.1 cells were not very sensitive to cell death by TRAIL (Fig. S1A), though they expressed high levels of DR4 and DR5 (Fig. 2A, B), which is likely explained by these cells being transformed by Epstein-Barr virus [31]. Clearly, stimulation of JeKo-1, RPMI8226 and BCWM.1 cells with various ER stress drugs did not reveal a crucial role for these receptors in cell death (Fig. 3A–C), independently of their basal expression. In all instances, cell death triggered by Thapsigargin, Bortezomib, or Brefeldin A proceeded also in absence of both receptors, with at best minor shifts in dose-response curves, e.g. in the Jeko-1 cells (Fig. 3A). These findings were confirmed by data from different clones of JeKo-1 and RPMI8226 DR5 KO and BCWM.1 DR4 KO (Fig. S3A–C). Even though JeKo-1 DR5 KO clones showed some clonal variation, the most deviant clone (KO clone #3) showed no significant role for the receptor (Fig. 3A). In comparison, in the lung adenocarcinoma cell line A549, which is insensitive to TRAIL treatment (Fig. 3D), removal of DR4 and DR5 (Fig. S2F) showed essential inhibition of cell death triggered by Thapsigargin, that relied mostly on DR5 and the DR4/5 together (Fig. 3D). A possible partial implication for DR4 was detected in JeKo-1 cells treated with Thapsigargin (Fig. 3A) which revealed significant inhibition of cell death upon DR4 KO at 1.6 nM and 3.1 nM (*p* < 0.005). In addition, upon treatment with Bortezomib slight dependency on DR5 and DR4/5 together was seen in JeKo-1 at 5, 10 nM (*p* ≤ 0.01), and at 20 nM only DR5 KO cells were significantly different (*p* = 0.01) (Fig. 3A). In summary, although DR4 and/or DR5 are essential for killing via TRAIL, they are dispensable for various forms of ER-stress induced-cell death in these B-cell malignancy cell lines.

### The intrinsic apoptosis pathway is involved in ER stress-cell death in B-cell malignancies

**Fig. 4:**
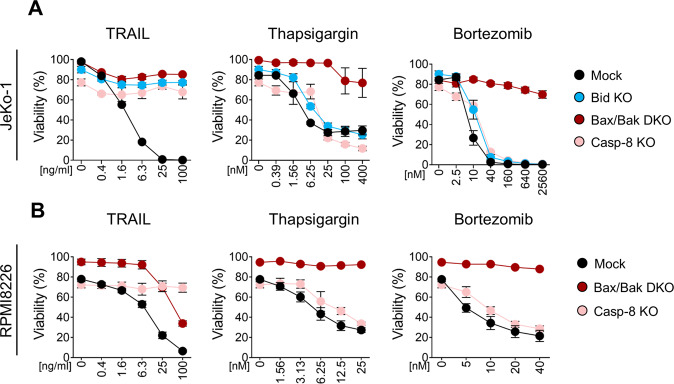
Consequences of Caspase-8, Bid and Bax/Bak gene ablation for TRAIL and ER-stress cell death responses. **A** JeKo-1 Mock, Bid KO (clonal population) and Casp-8 KO (clonal population) cells were incubated with TRAIL, Thapsigargin or Bortezomib for 24 hours and Bax/Bak DKO cells for 48 hours. Cells were then collected and viability was measured via FACS. Results are shown as Mean ± SEM (*N* = 3). **B** RPMI8226 Mock, Casp-8 KO (clonal population) and Bax/Bak DKO (clonal population) cells were incubated with TRAIL, Thapsigargin and Bortezomib with the indicated doses. After 24 hours incubation, cells were collected and viability measured via FACS by DiOC6 and ToPro-3 staining. Analyses were performed selecting cells for DiOC6^+^ and ToPro-3^-^. Results are shown as Mean ± SEM (*N* = 3).

**Fig. 5:**
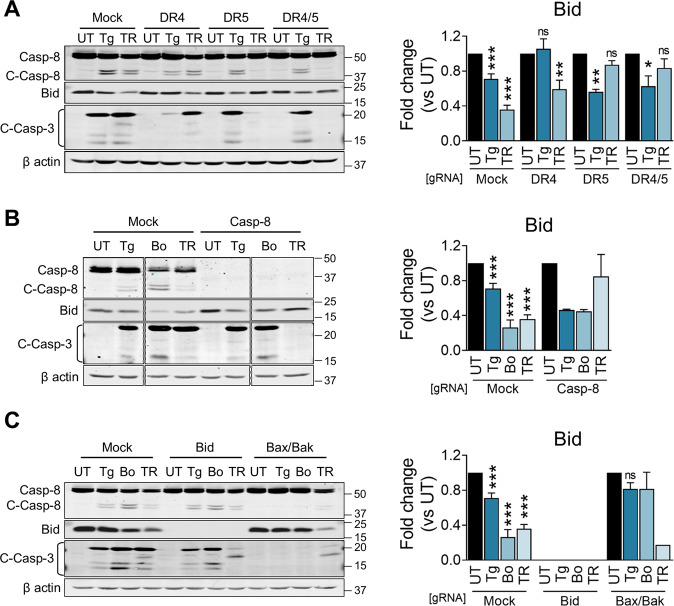
Consequences of Caspase-8, Bid and Bax/Bak gene ablation for TRAIL and ER-stress-induced Caspase-8/3 activation and Bid cleavage. **A** JeKo-1 Mock, DR4 KO (pool), DR5 KO (clonal population) and DR4/5 DKO (clonal population) cells were incubated with 20 nM Thapsigargin and 14 ng/ml TRAIL for 16 hours. Cells were then collected and proteins extracted. Caspase-8 (and its cleavage), Bid and Caspase-3 (and its cleavage) were measured via western blotting, using β-actin as loading control. Bid quantifications were performed using ImageJ and normalized to the untreated (UT) control for each KO. Results are shown as Mean ± SEM (*N* ≥ 3). Asterisks denote significant differences with their respectively untreated cells of each KO. **B** JeKo-1 Mock and Caspase-8 KO (clonal population) cells were incubated with 20 nM Thapsigargin, 40 nM Bortezomib and 14 ng/ml TRAIL for 16 hours. Cells were then collected and proteins extracted. Caspase-8 (and its cleavage), Bid and Caspase-3 (and its cleavage) were measured via western blotting, using β-actin as loading control. One membrane is shown, with cut-out intervening lanes. Bid quantifications were performed using ImageJ and normalized to the untreated (UT) control for each KO. Results are shown as Mean ± SEM (Mock N ≥ 4, Caspase-8 KO *N* = 2). **C** JeKo-1 Mock, Bid KO (clonal population) and Bax/Bak DKO (clonal population) cells were incubated with 20 nM Thapsigargin, 40 nM Bortezomib and 14 ng/ml TRAIL for 16 hours. Cells were then collected and proteins extracted. Caspase-8 (and its cleavage), Bid and Caspase-3 (and its cleavage) were measured via western blotting, using β-actin as loading control. Bid quantifications were performed using ImageJ and normalized to the untreated (UT) control for each KO. Results are shown as Mean ± SEM (Mock *N* ≥ 4, Bax/Bak DKO Thapsigargin N = 3 and Bortezomib and TRAIL *N* = 2).

To further investigate the mechanism by which B-cell malignancies undergo ER stress-mediated-cell death, the intrinsic versus extrinsic apoptosis pathway was taken into consideration. CRISPR/Cas9 KOs of Bid, Bax/Bak and Caspase-8 were created (Fig. S4A-C and Fig. 5B, C and [32] for Bax/Bak DKO in JeKo-1 cells) and ER stress induced-cell death measured. In JeKo-1 cells, treatment with TRAIL showed inhibition of cell death upon absence of Bax/Bak, Bid and Caspase-8 (Fig. 4A). In contrast, after stimulation with Thapsigargin or Bortezomib, JeKo-1 cells exhibited strong blockage of cell death only in absence of Bax/Bak, and at best minor reduction in Caspase-8 or Bid KOs at lower dosages. Similar results were seen in RPMI8226 cells, in which absence of Bax/Bak completely blocked cell death triggered by ER stress (Thapsigargin and Bortezomib) (Fig. 4B) while the single KO of either Bax or Bak had no effect (Fig. S4D). Notably, Bax/Bak DKO showed full resistance upon TRAIL stimulation in JeKo-1 and partial resistance in RPMI8226, which fits well with occurrence of so-called type I versus type II cells in their requirement of the mitochondrial pathway of apoptosis [33]. In addition, Caspase-8 KO prevented cell death only under TRAIL stimulation and not upon ER stress treatments (Fig. 4B). Data from BCWM.1 cells using several clones of Bid KO showed similar results as the JeKo-1 Bid KO, in which no inhibition was detected upon treatment with ER stress drugs (Fig. S4E). Together these data highlighted the essential role of Bax/Bak in ER stress-mediated-cell death, while Bid or Caspase-8 were dispensable, demonstrating that B-cell malignancies rely on the intrinsic rather than the extrinsic apoptosis process upon ER stress conditions.

### Crosstalk between Caspase-8 and the mitochondria in response to TRAIL and ER stress

The activation of the intrinsic cell death pathway and the potential involvement of crosstalk between the intrinsic and extrinsic apoptosis axis was investigated using JeKo-1 KOs cells (Fig. 5). In these cells, Caspase-3 and Bid cleavage corresponded to the data observed by earlier quantification of viability (Fig. 3A). TRAIL-induced caspase cleavage which was prevented by DR5 KO. DR4 KO partially prevented Caspase-8 cleavage by 20 nM Thapsigargin (Fig. 5A). Knocking out Caspase-8, showed a strong tendency in ablating Caspase-3 and Bid cleavage upon TRAIL treatment, but not upon ER stress induction (Fig. 5B). In addition, Bid KO showed no inhibition of cleavage of Caspase-8 and -3 upon ER stress, but did allow cleavage of the two caspases in response to TRAIL (Fig. 5C). Furthermore, Bax/Bak DKO fully abrogated Caspase-8, -3 and Bid cleavage with ER stimuli, and reduced these in response to TRAIL treatment. This, together with the data of Fig. 4A, B, indicates that Bax and Bak are essential proteins for ER stress induced-apoptosis and that cleavage of Caspase-8, -3 and Bid occurred after mitochondrial permeabilization. Moreover, this cleavage upon ER-stress was DR4/5 independent.

### Combined TRAIL and Thapsigargin, Tunicamycin or Bortezomib treatment shows synergistic effects in B-cell malignancies

**Fig. 6:**
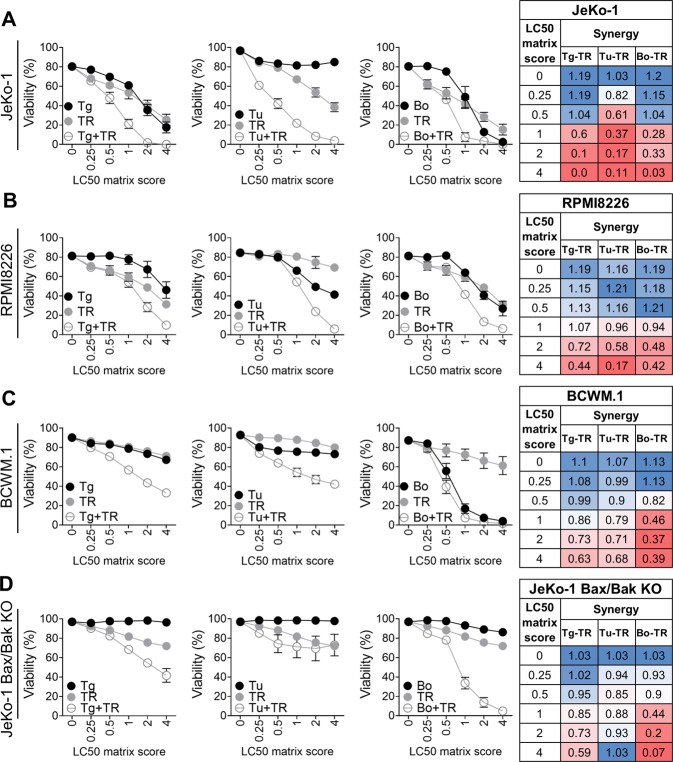
Synergy between TRAIL and ER-stress inducers in B cell lines. **A** JeKo-1 cells were incubated with Thapsigargin (Tg), Tunicamycin (Tu), Bortezomib (Bo), or TRAIL (TR) or the combination of the ER stress drugs with TRAIL for 24 hours. Cells were then collected and viability was measured via FACS using DiOC6 and ToPro-3 staining. Results are shown as Mean ± SEM (*N* = 3). Table shows synergy combination index scores calculated based on viability values of the curves. The scores in blue represents no synergy and in red strong synergy. **B** Same as A but with RPMI8226 cells. Results are shown as Mean ± SEM (*N* = 3). **C** Same as A but with BCWM.1 cells. Results are shown as Mean ± SEM (*N* = 3). **D** Same as A but with JeKo-1 Bax/Bak DKO (clonal population) cells. Results are shown as Mean ± SEM (*N* = 2).

The previous data demonstrated that DR4/5 are generally not involved in cell death triggered by ER-stress inducers. If the TRAIL and ER-stress death payhways are separate, this suggests that simultaneous triggering could be synergistic. Indeed, for the three B-cell malignancy cell lines tested, the addition of TRAIL to Thapsigargin,Tunicamycin or Bortezomib treatment had a positive synergistic effect (synergy value of < 0.8) in cell death already at low doses (Fig. 6A–C) (concentrations in Fig. S5A). Among the three cell lines, the strongest synergystic effect was observed in JeKo-1 cells, which were resistant to Tunicamycin treatment alone, whereas in combination with TRAIL showed synergism at low dosages. Notably, the BCWM.1 cells, which were insensitive to TRAIL, Thapsigargin or Tunicamycin, became sensitive to the combination (Fig. 6C). Furthermore, combining TRAIL with Thapsigargin and Bortezomib could even sensitize the resistant JeKo-1 Bax/Bak DKO cells, but not the combination of TRAIL with Tunicamycin (Fig. 6D). In conclusion, based on synergy scores we demonstrated a potential to sensitizing ER stress drugs with TRAIL treatment in several types of B-cell malignancy cell lines.

## Discussion

Apoptosis induced by the majority of drugs involves Bax, Bak and the mitochondrial pathway. Chemicals or physiological stimuli that perturb ER homeostasis are no exception [34], and a role for BIM as an universal mediator of cell death by ER stressors was proposed by studies in knock-out mice [9]. However, it was also reported that DRs, mainly DR5, are involved in ER stress-mediated cell death [4, 5, 26]. This has led to controversy and to the more recent realization that the role of DRs in ER-stress-mediated cell death could well be cell-type dependent [10, 29]. Increased sensitivity as well as the dependency of B-cells on ER function and the UPR, makes it an interesting subject in view of potential therapeutic applications for B-cell malignancies [12, 13, 23, 35]. In order to exploit the therapeutic potential of the UPR by redirecting it towards cell death, understanding which proteins are involved is essential.

In this study we showed that three B-cell malignancy cell lines from distinct origin were susceptible to four ER stress drugs, with some variation in sensitivity. As it is known that high expression of DR4 and/or DR5 can trigger the activation of the extrinsic apoptosis pathway in solid tumors, we delineated the basal and ER stress-induced expression of the receptors. We observed that at basal level their expression is cell-type dependent (Fig. 2A), and that mainly DR5 can be regulated upon ER stress, both at the transcriptional and translational level (Fig. 2C, D and Fig. S1C). Notably, all three cell lines when treated with Brefeldin A, showed a stronger increase of DR5 on the protein level, whereas it was not so much increased at the mRNA level. This could be explained by accumulation of DR5 in the ER as a result from inhibition of further protein transport to the Golgi and plasma membrane, where it would be shed and degraded after activation.

Engineered CRISPR/Cas9 KOs for both DRs in B-cell malignancy-derived cell lines confirmed their crucial role in TRAIL-mediated apoptosis. In contrast, under ER stress conditions both DR4 and DR5 were dispensable in triggering apoptosis (Fig. 3A–C). The minor shifts in cell death in DRs KO cells in JeKo-1 could in principle indicate partial involvement in ER stress death, but clearly DRs are not essential as was observed for solid tumors (Fig. 3D). Of note, although not all DR4/5 KO cell lines were 100% gene deleted (Fig. S2A-E), the data can still be interpreted with confidence, as previous studies have demonstrated that partial KO by siRNA already gave strong phenotypic responses [6]. Clearly, the KOs of Caspase-8 and Bid prevented TRAIL-mediated apoptosis, but this was not the case under ER stress conditions in B-cell malignancies (Fig. 4A, B). Only knocking out both Bax and Bak completely abrogated ER stress-induced cell death in B-cell malignancy cell lines. Although it is possible that both extrinsic and intrinsic cell death pathways cooperate, even within the same cell type [36], our data in JeKo-1 cells suggest the engagement of mainly intrinsic apoptosis for all stimuli. This was further confirmed in Bax/Bak DKO cells, which fully blocked cell death via TRAIL (Fig. 4A), defining these cells as type II cells that require the intrinsic pathway upon DRs triggering [33]. Interestingly, when JeKo-1 Bax/Bak DKO cells were treated with the combination of TRAIL and Thapsigargin and especially Bortezomib, they switched to type I cells and underwent apoptosis. On the other hand, RPMI8226 cells show a partial type I cell behavior since Bax/Bak DKO cells showed a shift towards reduced sensitivity to TRAIL (Fig. 4B). Overall, our data in the different KOs shows that while deficiency of the Caspase-8/Bid pathway prevented apoptosis upon TRAIL stimulation, ER stress still led to apoptosis. A small reduction in Caspase-8, -3 and Bid cleavage in DR4 KO JeKo-1 cells was observed, which correlates with the observed reduced cell death upon Thapsigargin treatment (Fig. 3A). Interestingly, Thapsigargin and Bortezomib led to Caspase-8 and Bid cleavage, but this did not occur in Bax/Bak deficient cells (Fig. 5), strongly suggesting that this is due to post-mitochondrial Caspase-3 activation, as both Caspase-8 and Bid are substrates of Caspase-3 [37]. In response to TRAIL, in absence of Bid or Bax/Bak, we observed incomplete cleavage of Caspase-3, probably because Bax/Bak oligomerization, cytochrome c and Smac release, and subsequent XIAP neutralization could be needed to fully cleave Caspase-3 into all its subunits in type II cells for death receptors [25].

Multiple previous reports demonstrated that triggering the extrinsic [38] and intrinsic apoptosis pathway together, results in a synergistic effect in solid tumors [39–41]. Therefore, we applied the same concept to the B-cell malignancy models and tested the sensitivity of TRAIL together with ER stress induction. In all three cell lines, the combination led to a synergistic effect. Even using Tunicamycin, synergy with TRAIL was observed, while cells were hardly sensitive to single treatment with Tunicamycin. With respect to therapeutic application, various clinical trials have explored TRAIL and TRAIL receptor agonistic antibodies, though these have not been successful [42]. In recent years, some phase I studies have investigated anti-tumor effects of second-generation TRAIL receptor agonists like IGM-8444 and ABBV-621/APG880 in hematological malignancies, although results are not yet available (For a review see [43]). The search for drugs that would specifically sensitize cancer cells to TRAIL analogs continues, and our data suggest that for B-cell malignancies, agents that induce ER stress could be part of the arsenal.

In conclusion, in the present study we delineated the cell death mechanism induced by ER stress in B-cell malignancies. We demonstrate a dispensable role for the DR4 and 5 in ER stress-induced cell death in these cell lines.

## Materials and methods

### Cell lines and treatments

JeKo-1 cells (obtained from DSMZ) and BCWM.1 (kindly provided by Dr. S.P. Treon to Dr. M. Spaargaren) were cultured in RPMI-1640 (ThermoFisher Scientific, Massachusetts, United States), RPMI8226 cells (obtained from ATCC) were cultured in IMDM (ThermoFisher Scientific) and A549 cells were cultured in High-glucose DMEM (ThermoFisher Scientific) at 37 °C and 5% CO_2_. Media were supplemented with 10% fetal calf serum (FCS) and 1% Penicillin-Streptomycin. Cell lines were authenticated by short tandem repeat analysis.

Drugs used to induce ER stress: Thapsigargin (Sigma Aldrich, Missouri, United States), Tunicamycin (Calbiochem, California, United States), Brefeldin A (Merck, New Jersey, United States), Bortezomib (Santa Cruz, California, United States). As positive control for DRs signalling, super-killer TRAIL (Enzo Life Sciences, New York, United States) was used. Concentrations used to induce ER stress and DR expression were aimed to induce a strong stress response without leading to full cell death and were derived from dose-response curves. Synergy experiments were performed using an LC50 matrix, which was based on calculated LC50 values using a linear regression model function from GraphPad Prism software (Version 9.1.0). Concentrations are shown in Supplementary Figure 5A. Synergy combination index scores were calculated as described in supplementary material elsewhere [44].

### Generation of CRISPR-Cas9 knock-out cell lines

KO cell lines for Bax, Bak, Bax/Bak, Bid, Casp-8 and DR5 were generated as previously described [32], by cloning gRNAs into the lentiCRISPRv2-puro plasmid (Addgene #98290) and by transduction with lentivirus. Transduced cells were selected using Puromycin (1 μg/ml) to obtain a pool of KO cells. In the case of a partial KO of the pool, limiting dilution was performed to generate a population of a single KO clone, unless clones were not viable. For DR4 KO, target cells were first transduced with Cas9 protein, followed by the transduction with DR4 in lentiGuide-dTomato-hygro plasmid (Addgene #99376). Transduced cells were selected using 1 mg/ml hygromycin. To generate DR4/5 DKO, a clonal population of DR5 KO was used to subsequently transduce it with DR4 gRNAs. All KOs were verified by western blot, and by FACS for the detection of DRs on cell surface. In JeKo-1 cells a KO pool was used for DR4, and clonal populations were used for DR5, DR4/5, Bax/Bak DKO, Caspase-8 and Bid. In RPMI8226 cells KO pools were used for DR4 and DR4/5 DKO, and clonal populations for DR5, Bax/Bak DKO and Caspase-8. In BCWM.1 cells a KO pool was used for DR4/5 DKO, and clonal populations for DR4, DR5, DR4/5 and Bid clones. In A549 cells a KO pool was used for DR4, and clonal populations were used for DR5 KO and DR4/5 DKO.

### Flow cytometry

Cell viability was assessed using DiOC6 (ThermoFisher Scientific) and ToPro-3 (ThermoFisher Scientific) staining and measured on FACS-Calibur (BD Biosciences, California, United States). Cells were stained 30 min at 37 °C with DiOC6, followed by incubation with ToPro-3 for 10 min. Data were analyzed using FlowJo v10 software and viable cells were considered to be DiOC6^+^ and ToPro-3^-^. For DR4 and DR5 surface stainings cells were washed phosphate-buffered saline (PBA; 0.5% BSA and 0.02% sodium azide) and incubated with fluorescently labeled antibodies for 20 min at 4 °C. A list of antibodies is provided in supplementary Table 1.

### Western blot

Protein lysates were generated using RIPA buffer, and after SDS-page were transferred onto PVDF membrane (B-cell malignancies blots) or nitrocellulose (A549 blots). Membranes were either developed using the Odyssey Imager (Li-Cor Biosciences, Nebraska, United States) or with the ImageQuant LAS 4000 mini using Pierce™ ECL Western Blotting Substrate (ThermoFisher Scientific). DR4, DR5 and Bid expression were quantified using ImageJ (Version 1.50i) software and normalized to β-actin expression. A list of antibodies is provided in supplementary Table 1.

### Gene expression analysis

Gene expression was measured as described before [45]. Only 1.3 μM of forward and reverse primer mixture and the PowerUp SYBER Green Fast Start Master mix (Applied Biosystems, Invitrogen, Massachusetts, United States) were used. Quantitation cycles were determined with Quantstudio3 (ThermoFisher Scientific) and then normalized to TBP as internal control. Fold change was calculated relative to the untreated sample. A list of oligonucleotides is provided in supplementary Table 2.

### Statistics

*P* values were calculated using One-way ANOVA in combination with Šídák’s multiple comparisons tests. Differences were considered significant when *p* < 0.05 (*), *p* < 0.01 (**) or *p* < 0.001 (***).

Supplementary Information accompanies the paper on the Oncogenesis website (http://www.nature.com/oncsis).

## Supplementary information

Figure S1. Quantification of viability and death receptor expression of cells treated with TRAIL and ER stress inducers

Figure S2. Confirmation of DR CRISPR/Cas-9 KOs in cell lines

Figure S3. Additional cell death curves to asses clonal variation of several KO clones

Figure S4. Additional cell death curves in RPMI8226 and BCWM.1 cells and validation of cell knock-outs

Figure S5. Concentrations of individual drugs in LC50 matrix scores

Antibody List

Oligonucleotide List

## Data Availability

All data generated or analysed during this study are included in this published article (and its supplementary information files).

## References

[1] Walter P, Ron D (2011). The unfolded protein response: from stress pathway to homeostatic regulation. Science.

[2] Iurlaro R, Munoz-Pinedo C (2016). Cell death induced by endoplasmic reticulum stress. FEBS J.

[3] Kraus M, Ruckrich T, Reich M, Gogel J, Beck A, Kammer W (2007). Activity patterns of proteasome subunits reflect bortezomib sensitivity of hematologic malignancies and are variable in primary human leukemia cells. Leukemia.

[4] Lu M, Lawrence DA, Marsters S, Acosta-Alvear D, Kimmig P, Mendez AS (2014). Opposing unfolded-protein-response signals converge on death receptor 5 to control apoptosis. Science.

[5] Yamaguchi H, Wang HG (2004). CHOP is involved in endoplasmic reticulum stress-induced apoptosis by enhancing DR5 expression in human carcinoma cells. J Biol Chem.

[6] Iurlaro R, Puschel F, Leon-Annicchiarico CL, O’Connor H, Martin SJ, Palou-Gramon D (2017). Glucose Deprivation Induces ATF4-Mediated Apoptosis through TRAIL Death Receptors. Mol Cell Biol.

[7] Wang Q, Mora-Jensen H, Weniger MA, Perez-Galan P, Wolford C, Hai T (2009). ERAD inhibitors integrate ER stress with an epigenetic mechanism to activate BH3-only protein NOXA in cancer cells. Proc Natl Acad Sci USA.

[8] Ramirez-Peinado S, Alcazar-Limones F, Lagares-Tena L, El Mjiyad N, Caro-Maldonado A, Tirado OM (2011). 2-deoxyglucose induces Noxa-dependent apoptosis in alveolar rhabdomyosarcoma. Cancer Res.

[9] Puthalakath H, O’Reilly LA, Gunn P, Lee L, Kelly PN, Huntington ND (2007). ER stress triggers apoptosis by activating BH3-only protein Bim. Cell.

[10] Glab JA, Doerflinger M, Nedeva C, Jose I, Mbogo GW, Paton JC (2017). DR5 and caspase-8 are dispensable in ER stress-induced apoptosis. Cell Death Differ.

[11] Todd DJ, Lee AH, Glimcher LH (2008). The endoplasmic reticulum stress response in immunity and autoimmunity. Nat Rev Immunol.

[12] Michallet AS, Mondiere P, Taillardet M, Leverrier Y, Genestier L, Defrance T (2011). Compromising the unfolded protein response induces autophagy-mediated cell death in multiple myeloma cells. PLoS One.

[13] Molinari M, Sitia R (2005). The secretory capacity of a cell depends on the efficiency of endoplasmic reticulum-associated degradation. Curr Top Microbiol Immunol.

[14] Maestre L, Tooze R, Canamero M, Montes-Moreno S, Ramos R, Doody G (2009). Expression pattern of XBP1(S) in human B-cell lymphomas. Haematologica.

[15] Shaffer AL, Wright G, Yang L, Powell J, Ngo V, Lamy L (2006). A library of gene expression signatures to illuminate normal and pathological lymphoid biology. Immunol Rev.

[16] Mimura N, Fulciniti M, Gorgun G, Tai YT, Cirstea D, Santo L (2012). Blockade of XBP1 splicing by inhibition of IRE1alpha is a promising therapeutic option in multiple myeloma. Blood.

[17] Nikesitch N, Lee JM, Ling S, Roberts TL (2018). Endoplasmic reticulum stress in the development of multiple myeloma and drug resistance. Clin Transl Immunol.

[18] Carrasco DR, Sukhdeo K, Protopopova M, Sinha R, Enos M, Carrasco DE (2007). The differentiation and stress response factor XBP-1 drives multiple myeloma pathogenesis. Cancer Cell.

[19] Robak P, Robak T (2019). Bortezomib for the Treatment of Hematologic Malignancies: 15 Years Later. Drugs R D.

[20] Treon SP, Hunter ZR, Matous J, Joyce RM, Mannion B, Advani R (2007). Multicenter clinical trial of bortezomib in relapsed/refractory Waldenstrom’s macroglobulinemia: results of WMCTG Trial 03-248. Clin Cancer Res.

[21] Ri M, Iida S, Nakashima T, Miyazaki H, Mori F, Ito A (2010). Bortezomib-resistant myeloma cell lines: a role for mutated PSMB5 in preventing the accumulation of unfolded proteins and fatal ER stress. Leukemia.

[22] Davenport EL, Moore HE, Dunlop AS, Sharp SY, Workman P, Morgan GJ (2007). Heat shock protein inhibition is associated with activation of the unfolded protein response pathway in myeloma plasma cells. Blood.

[23] Obeng EA, Carlson LM, Gutman DM, Harrington WJ, Lee KP, Boise LH (2006). Proteasome inhibitors induce a terminal unfolded protein response in multiple myeloma cells. Blood.

[24] Liu X, Kim CN, Yang J, Jemmerson R, Wang X (1996). Induction of apoptotic program in cell-free extracts: requirement for dATP and cytochrome c. Cell.

[25] Jost PJ, Grabow S, Gray D, McKenzie MD, Nachbur U, Huang DC (2009). XIAP discriminates between type I and type II FAS-induced apoptosis. Nature.

[26] Martin-Perez R, Palacios C, Yerbes R, Cano-Gonzalez A, Iglesias-Serret D, Gil J (2014). Activated ERBB2/HER2 licenses sensitivity to apoptosis upon endoplasmic reticulum stress through a PERK-dependent pathway. Cancer Res.

[27] Lam M, Marsters SA, Ashkenazi A, Walter P (2020). Misfolded proteins bind and activate death receptor 5 to trigger apoptosis during unresolved endoplasmic reticulum stress. Elife.

[28] McGrath EP, Centonze FG, Chevet E, Avril T, Lafont E (2021). Death sentence: The tale of a fallen endoplasmic reticulum. Biochim Biophys Acta Mol Cell Res.

[29] Munoz-Pinedo C, Lopez-Rivas A (2018). A role for caspase-8 and TRAIL-R2/DR5 in ER-stress-induced apoptosis. Cell Death Differ.

[30] Mora-Molina R, Stohr D, Rehm M, Lopez-Rivas A (2022). cFLIP downregulation is an early event required for endoplasmic reticulum stress-induced apoptosis in tumor cells. Cell Death Dis.

[31] Snow AL, Lambert SL, Natkunam Y, Esquivel CO, Krams SM, Martinez OM (2006). EBV can protect latently infected B cell lymphomas from death receptor-induced apoptosis. J Immunol.

[32] Martens AWJ, Janssen SR, Derks IAM, Adams HC, Izhak L, van Kampen R (2020). CD3xCD19 DART molecule treatment induces non-apoptotic killing and is efficient against high-risk chemotherapy and venetoclax-resistant chronic lymphocytic leukemia cells. J Immunother Cancer.

[33] Scaffidi C, Fulda S, Srinivasan A, Friesen C, Li F, Tomaselli KJ (1998). Two CD95 (APO-1/Fas) signaling pathways. EMBO J.

[34] Zong WX, Lindsten T, Ross AJ, MacGregor GR, Thompson CB (2001). BH3-only proteins that bind pro-survival Bcl-2 family members fail to induce apoptosis in the absence of Bax and Bak. Genes Dev.

[35] Gomez-Bougie P, Halliez M, Moreau P, Pellat-Deceunynck C, Amiot M (2016). Repression of Mcl-1 and disruption of the Mcl-1/Bak interaction in myeloma cells couple ER stress to mitochondrial apoptosis. Cancer Lett.

[36] Cano-Gonzalez A, Mauro-Lizcano M, Iglesias-Serret D, Gil J, Lopez-Rivas A (2018). Involvement of both caspase-8 and Noxa-activated pathways in endoplasmic reticulum stress-induced apoptosis in triple-negative breast tumor cells. Cell Death Dis.

[37] Slee EA, Keogh SA, Martin SJ (2000). Cleavage of BID during cytotoxic drug and UV radiation-induced apoptosis occurs downstream of the point of Bcl-2 action and is catalysed by caspase-3: a potential feedback loop for amplification of apoptosis-associated mitochondrial cytochrome c release. Cell Death Differ.

[38] Nahacka Z, Svadlenka J, Peterka M, Ksandrova M, Benesova S, Neuzil J (2018). TRAIL induces apoptosis but not necroptosis in colorectal and pancreatic cancer cells preferentially via the TRAIL-R2/DR5 receptor. Biochim Biophys Acta Mol Cell Res.

[39] Lee SJ, Lee DE, Choi SY, Kwon OS (2021). OSMI-1 Enhances TRAIL-Induced Apoptosis through ER Stress and NF-kappaB Signaling in Colon Cancer Cells. Int J Mol Sci.

[40] Vincenz L, Jager R, O’Dwyer M, Samali A (2013). Endoplasmic reticulum stress and the unfolded protein response: targeting the Achilles heel of multiple myeloma. Mol Cancer Ther.

[41] White-Gilbertson S, Hua Y, Liu B (2013). The role of endoplasmic reticulum stress in maintaining and targeting multiple myeloma: a double-edged sword of adaptation and apoptosis. Front Genet.

[42] von Karstedt S, Montinaro A, Walczak H (2017). Exploring the TRAILs less travelled: TRAIL in cancer biology and therapy. Nat Rev Cancer.

[43] Montinaro A, Walczak H. Harnessing TRAIL-induced cell death for cancer therapy: a long walk with thrilling discoveries. Cell Death Differ. 2022. 10.1038/s41418-022-01059-z 10.1038/s41418-022-01059-z PMC9950482 36195672

[44] Haselager MV, Kielbassa K, Ter Burg J, Bax DJC, Fernandes SM, Borst J (2020). Changes in Bcl-2 members after ibrutinib or venetoclax uncover functional hierarchy in determining resistance to venetoclax in CLL. Blood.

[45] Puschel F, Favaro F, Redondo-Pedraza J, Lucendo E, Iurlaro R, Marchetti S (2020). Starvation and antimetabolic therapy promote cytokine release and recruitment of immune cells. Proc Natl Acad Sci USA.

