# Supplementary figures and images for: Negligible role of TRAIL death receptors in cell death upon endoplasmic reticulum stress in B-cell malignancies
Source: Oncogenesis. 2023 Feb 8;12(1):6. doi: 10.1038/s41389-023-00450-w (PMC9908905; doi:10.1038/s41389-023-00450-w)

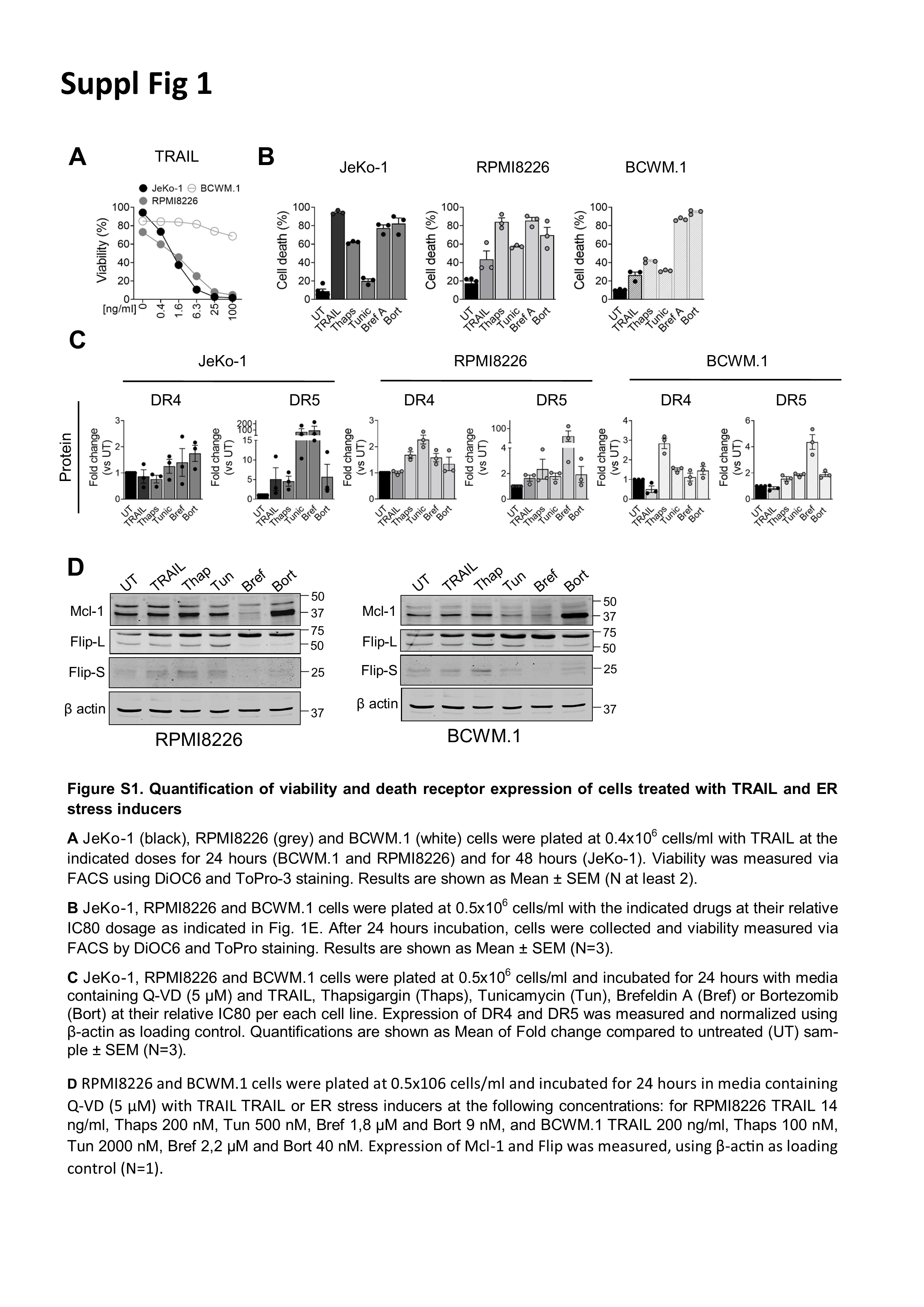

Supplement: Supplementary file 1 — Figure S1. Quantification of viability and death receptor expression of cells treated with TRAIL and ER stress inducers [file 41389_2023_450_MOESM1_ESM.tif]

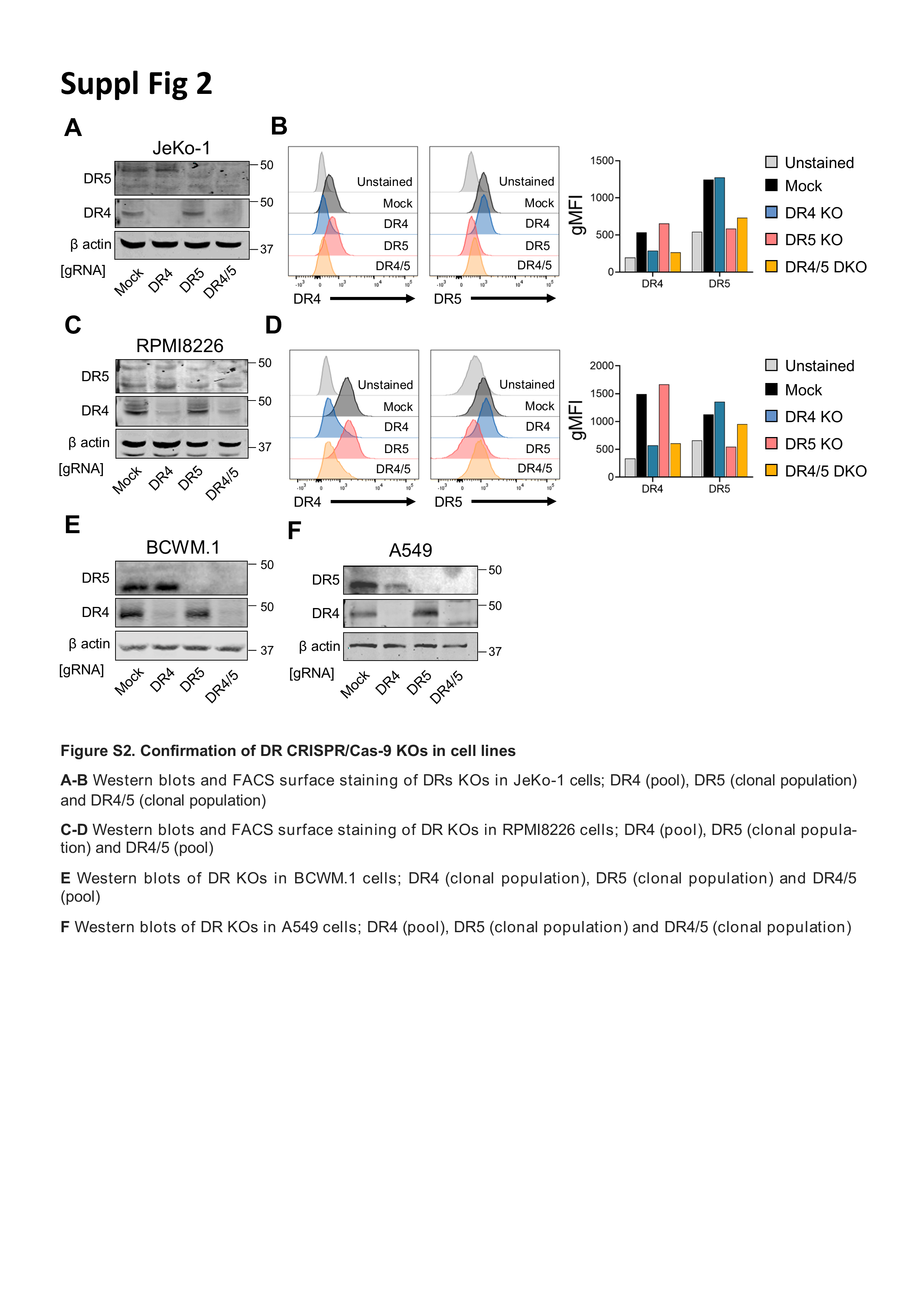

Supplement: Supplementary file 2 — Figure S2. Confirmation of DR CRISPR/Cas-9 KOs in cell lines [file 41389_2023_450_MOESM2_ESM.tif]

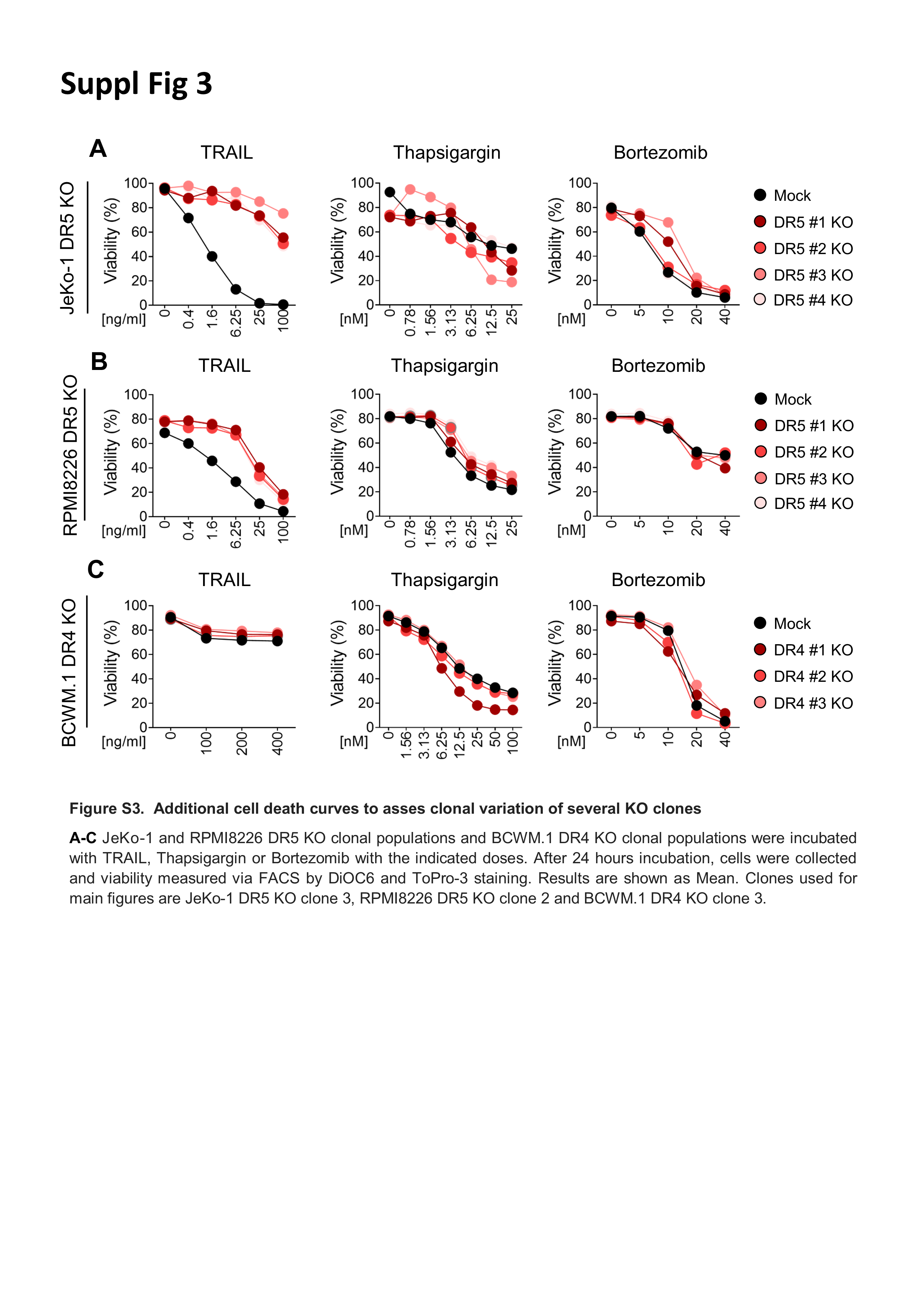

Supplement: Supplementary file 3 — Figure S3. Additional cell death curves to asses clonal variation of several KO clones [file 41389_2023_450_MOESM3_ESM.tif]

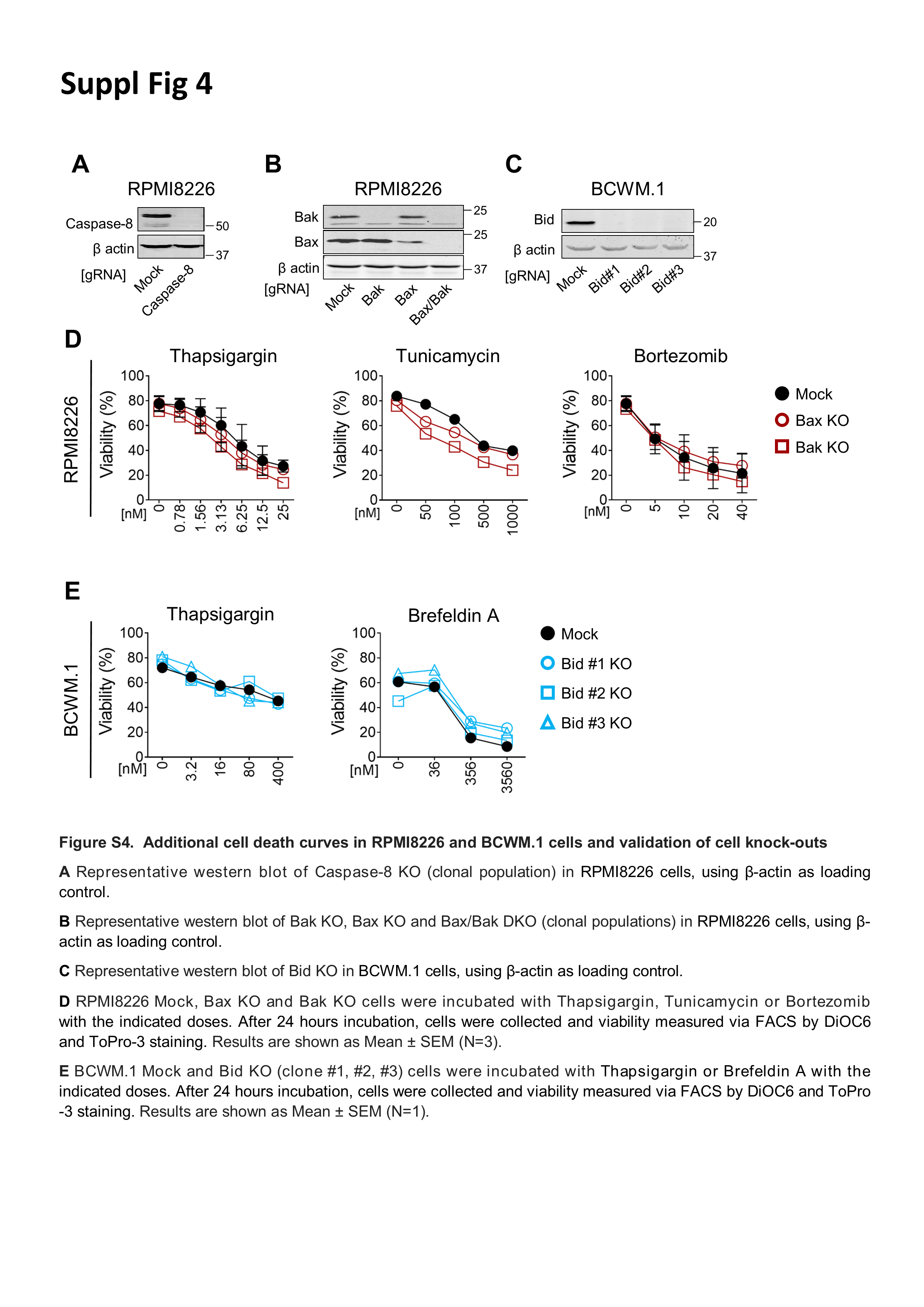

Supplement: Supplementary file 4 — Figure S4. Additional cell death curves in RPMI8226 and BCWM.1 cells and validation of cell knock-outs [file 41389_2023_450_MOESM4_ESM.tif]

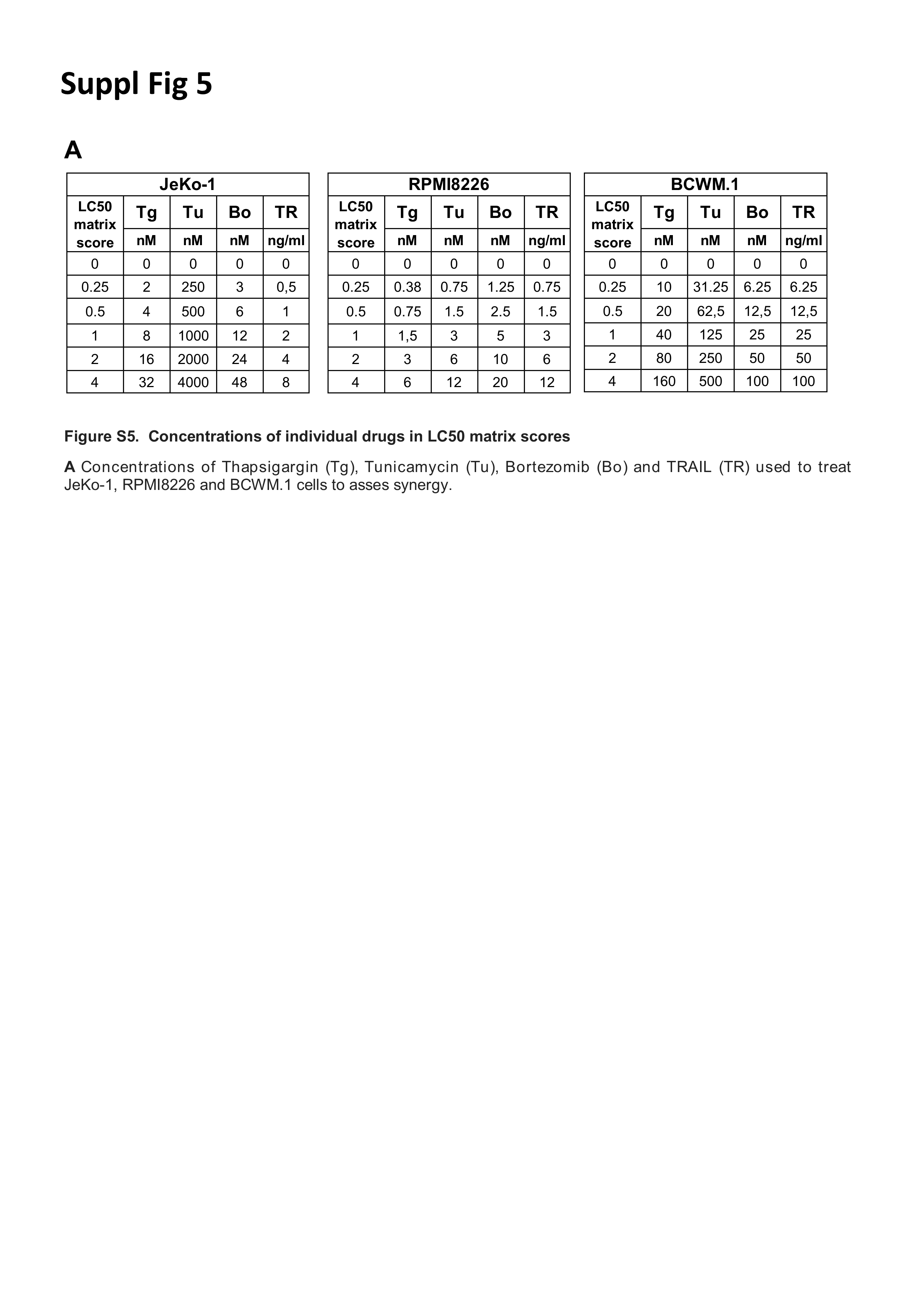

Supplement: Supplementary file 5 — Figure S5. Concentrations of individual drugs in LC50 matrix scores [file 41389_2023_450_MOESM5_ESM.tif]
